# Jefferson Medical College

**Published:** 1864-04

**Authors:** 


Jefferson Medical College.—The number of students during the past season was 351; and at the commencement held on the 10th of March the degree of M. D, was conferred on 124 candidates.

